# Adaptation of a model for integration of interventions for alcohol use disorders in primary health care in Tanzania

**DOI:** 10.1186/s12875-023-02061-1

**Published:** 2023-04-21

**Authors:** Dorothy Mushi, Charlotte Hanlon, Joel M. Francis, Moshiro Candida, Mekdes Demissie, Solomon Teferra

**Affiliations:** 1 Department of Psychiatry and Mental Health, Muhimbili University of Health and Allied Science, P.O Box 65001, United Nations Road, Dar Es Salaam, Tanzania; 2 Department of Psychiatry, School of Medicine, College of Health Sciences, Addis Ababa University, Addis Ababa, Ethiopia; 3 Centre for Innovative Drug Development and Therapeutic Trials for Africa (CDT-Africa), College of Health Science, Addis Ababa University, Addis Ababa, Ethiopia; 4 Centre for Global Mental Health, Health Service and Population Research Department, Institute of Psychiatry, Psychology, and Neuroscience, King’s College London, London, UK; 5 Department of Family Medicine and Primary Care, Witwatersrand University, Faculty of Health Sciences, Johannesburg, South Africa; 6 Department of Epidemiology and Biostatistics, Muhimbili University of Health and Allied Sciences, Dar Es Salaam, Tanzania; 7 Psychiatric Nursing Department, School of Nursing and Midwifery, College of Health and medical Sciences, Haramaya University, Ethiopia, Dire Dawa , Ethiopia

**Keywords:** Alcohol use disorders, Adaptation, Intervention packages, Integration, Primary health care, Tanzania

## Abstract

**Background:**

Integrating evidence-based interventions for people with alcohol use disorder (AUD) into primary healthcare (PHC) can increase access to care and reduce morbidity, mortality, and population burden. However, for the integration to be feasible, acceptable, and sustainable, there is a need to contextualize the approach and involve stakeholders. Therefore, this study aimed to use participatory methods to adapt a model for integrating AUD interventions in Tanzania’s PHC system at the community, facility, and organizational levels.

**Methods:**

A mixed-methods study was used. Participants include key mental health stakeholders, experts, and PHC providers. We conducted a situational analysis to investigate opportunities and constraints in the existing systems of care, utilizing data available from the routine collection and/or in the public domain, individual semi-structured interviews (*n* = 11), and focus group discussions (3 groups; total *n* = 26 participants) and a series of theory of change (ToC) workshops (*n* = 32). Data from the three methods were triangulated to develop the adapted model for integrating interventions for AUD in PHC.

**Results:**

A situational appraisal revealed limited community, facility, and organizational resources and infrastructures for supporting services delivery of integrated AUD interventions. Also, shortage of health workforce, inadequate health management information systems, and limited medical supply and financing. Nevertheless, the theory of change proposed integrated AUD intervention packages and strategies to facilitate integrated care for people with AUD. Additionally, the barriers and facilitators for implementing these integrated AUD interventions and how to overcome them were explored.

**Conclusions:**

The adapted model for the integrated AUD intervention in Tanzanian PHC revealed limited resources and system functioning for facilitating integrated AUD services. Nevertheless, it proposes the needed integrated AUD interventions and its barriers, facilitators, and strategies for overcoming them. There is a need to pilot the adapted model to inform plans for more comprehensive implementation or scaling up.

## Background

Alcohol use disorder (AUD) is characterized by an impaired ability to stop or control alcohol use despite adverse social, occupational, or health consequences [1]. In 2016, the World Health Organization’s (WHO) Global Status Report on Alcohol and Health reported that harmful use of alcohol caused about 5.1% of all disability-adjusted life years (DALYs) and accounts for three million of all death annually which is 5.3% of all deaths [2]

In sub-Saharan Africa (SSA), approximately one in five people attending healthcare facilities meet the criteria for AUD [3–7]. Alcohol use and AUD have been associated with poor clinical and psychosocial outcomes, including depression, stigma, disability, and risky sexual behaviors [8–11]. In Tanzania, among people attending PHC facilities, 60.7% reported alcohol use, heavy episodic drinking was reported by 37.3%, and AUD (AUD Identification Test (AUDIT) ≥ 8) was present in 23.9%. (PHC) [12].

Despite AUD's magnitude and negative consequences, the problem is not commonly detected or treated in health facilities [11–16]. In keeping with this, our formative scoping review study on the integration of AUD interventions in SSA revealed limited evidence on the integration of AUD interventions in healthcare settings in SSA [17]. In addition, low help-seeking behavior and barriers to care for AUD services are substantial [11, 12, 18]. Therefore, the treatment gap for AUD is wide [19], particularly in low- and middle-income countries (LMICs) [11, 13, 14, 16].

To narrow this treatment gap for AUD, the WHO has identified evidence-based interventions for priority mental, neurological, and substance use disorders (MNS) that can be integrated into PHC and other non-specialized settings such as comprehensive policy measures and legislative interventions, screening and brief interventions, training PHC, early identification and treatment of AUD in PHC, referral and supervisory [19]. However, adaptation is essential to ensure that the delivery mechanisms and content of these packages of care are effective, sustainable, acceptable, and feasible in different socio-cultural and health system contexts [20–22]. In response, some SSA countries have developed and implemented mental health care plans, frameworks, and programs for integrating mental health care in PHC [20, 23, 24]. There is increasing evidence that implementing these integrated approaches to mental health care in SSA contexts can improve clinical and psychosocial outcomes for people with mental health and substance use conditions, including those with AUD [25].

The Tanzanian Ministry of Health (MoH) seeks to integrate mental health care into non-specialist healthcare platforms, as specified in the National health sector strategic plan V and the national strategic plan for non-communicable diseases [26, 27]. Yet our facility-based survey in the study setting found that, out of 378 participants with a probable AUD (AUDIT score ≥ 8), screening and management of AUD were recorded for only one participant. Only 5% (20/378) had reported seeking help from informal and non-informal sources, and substantial barriers to seeking care were reported [12].

Therefore, developing a contextually relevant plan for integrated care is essential to address these gaps. The current study aimed to adapt a model for integrating AUD interventions into the Tanzania PHC system.

## Methods

### Study design

The study protocol has been published previously [28]. We used a mixed methods approach to adapt a model for integrating AUD interventions: (i) a cross-sectional situational analysis to understand resources and system functioning in relation to the proposed integration of services for AUD in PHC in the study area, (ii) Theory of Change (ToC), a participatory approach that aimed to map out how integrated AUD care can be implemented to achieve its goal, and (iii) a qualitative study to explore feasibility and acceptability of the adapted integrated care model for AUD in PHC and identify facilitators, barriers, and implementation strategies Table 1.

**Table 1:** Methods of studies to inform adaptation of a model for integration of AUD interventions

Method		Summary of findings	Contribution to the adaptation of a model for integration of interventions for AUD
1	Scoping review [17]	Identified interventions packages and strategies Informed methods	Defined the type of intervention at a different level Defined the intervention content
2	A cross-sectional facility survey [12])	The magnitude of AUD among people attending PHC was 23.9%, and the PHC workers detected only 0.3% to have AUD. People with AUD had low help-seeking behavior and substantial barriers to seeking care	Informed unmet need to care for people with AUD
3	Situational analysis	Mapped available and needed resources for supporting the integration of AUD services in PHC	Understand the context and available resources
4	ToC workshops	Possible interventions components Need for training for HCPs and CHWs Need to improve recruitment of staff Need to strengthen health financing Need to improve medical infrastructure Need for multisectoral engagement	Explored the feasibility and acceptability of the model for integration Defined the necessary resources, outcomes, and causal pathway Defined indicators for success
5	Qualitative	Facilitators include the existence of health care resources and capacity building of HCP and CHWs Barriers to shortage of staff Strategies to improve health financing and staff recruitment	Explored the feasibility of acceptability, anticipated barriers, and strategies to overcome them

### Study setting

**Fig. 1:**
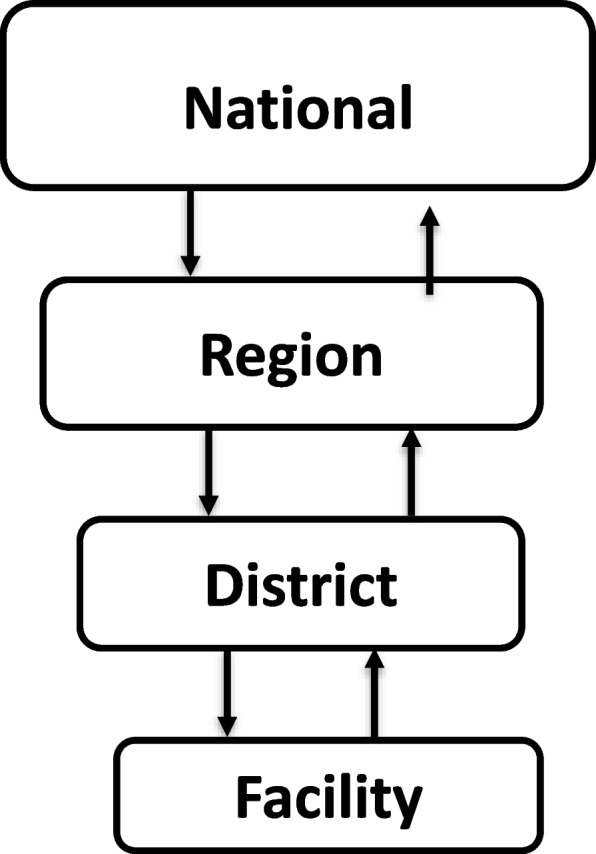
Tanzanian health services organization

The study setting was the Moshi district council, one of the seven administrative districts in the Kilimanjaro region, which is located around 530 km from the main economic city of Dar es Salaam (the former capital city of Tanzania). Tanzanian administration is organized at the local government, including (village/street, → ward, → sub-division, → district, → region) and central. The Moshi district council has 88 dispensaries, eight health centers (six are government and two private), and two district hospitals. Dispensaries are the first contact point for basic health services in the community. Tanzania’s health system is organized (refers to aspects of leadership /administrative/coordinating) at facility, district, regional, and national levels (Fig. 1). However, health services are provided through the dispensary, health centers, and district, regional, and tertiary hospitals. The first three levels (dispensary, health center, and district) constitute the Tanzanian PHC system, in which the adapted model will guide the integration of AUD interventions in general healthcare services.

The study participants were health services implementers and administrators/managers from the Moshi district council. Also, health administrators/ managers at regional and national. The participants were selected purposively based on their mental health service provision roles. We involve HCPs and heads of facilities from all six government health centers, local government, women leaders, the district health management team, the regional health management team, and the national mental health coordinator. More details have been provided in a particular data collection method.

The study area was selected based on studies of AUD in Tanzania [29, 30]. In these studies, alcohol use (68.0 to 70.0%) and problematic alcohol use (20.0 to 47.0%) were found to be highly prevalent in the community [29, 30]. Based on previous findings, it was recommended that access to interventions for AUD should be expanded [29–32]. Therefore, this study builds on formative studies to adapt a model for integrating care for people with AUD in PHC in the study area [12, 17, 28].

### Adaptation of an AUD model

The WHO packages evidence-based interventions for priority MNS conditions [19], including (comprehensive policy measures and legislative interventions, screening and brief interventions, training PHC, early identification and treatment of AUD in PHC, referral, and supervisory). Also, the mental health plans, frameworks, and programs for the integration of MHS that were developed and implemented in a nearby Eastern African country [20] and other SSA countries [23, 24] informed the contents and methods for the adaptation of the model for integration of interventions for AUD in Tanzanian PHC. See Table 2. Firstly, the study team presented to the participants in the ToC meetings the objective of the study, findings of situational analysis, contents and methods of the WHO intervention packages, the mental health plans, frameworks, and programs that inform the method and contents to be used for the adaptation of the model for the integration of AUD intervention in Tanzanian PHC. The presentation facilitates participants’ familiarization and discussion engagement for adapting the Tanzanian model for integrating AUD intervention in PHC. Below is a description of each method.

**Table 2:** Summary of the previous program and plan that informed the adapted model

Program/plan	Content	Method
Mental Health Gap Action Programme (mhGAP) and the mhGAP Intervention Guide (mhGAP-IG) [19]	Evidence-based facility and organization interventions (including comprehensive policy measures and legislative interventions. Screening and brief interventions by trained primary healthcare professionals. Early identification and treatment of AUD in PHC. Referral and supervisory	
The development of the district mental health plan in Ethiopia [20] and in South Africa [23]	Mental health interventions packages and expected short outcomes at community, facilities, and organization	Situational appraisal Theory of change Qualitative study
Project for integrating MH in Nigeria [24]	Training and supervision to health care providers for integrating mental health	

### Situational analysis

A situational analysis tool [33] was adapted to in-keep with the Tanzanian healthcare system organization structure. Therefore, to collect information on resources and system functioning with respect to the potential integration of interventions for people with AUD in PHC in the Moshi district council. A more detailed is provided in the protocol [28]. The tool was designed for district-level care planning for mental health and substance use disorders in LMICs. The tool has been applied previously at the district and sub-district levels in two other countries in East Africa [20, 23]. The tool provides a structured format for systematically mapping the available resources and constraints to integrating services for people with MNS, including AUD.

The study coordinator, a psychiatrist, administered the situation analysis tool to responsible personnel at the community (community outreach coordinator), facility (heads of the facilities), and organization levels (district, Regional, and National mental health coordinators). The information collected at community, facilities, and organizations informed different health system pillars, including leadership, financing, human resources, services, monitoring and evaluation, and medication/psychosocial interventions. In addition, contextual information, such as community resources. The tool relies on information in the public domain and consultation with key officials or public figures.

Data were collected from August to December 2019. Responsible personnel was contacted if data needed to be more consistent or complete. The quantitative information collected from the situation analysis was summarized descriptively (using frequencies), yielding information on the number of HCPs (by category, specialization, e.g., mental health and non-mental health) and (ii) the number of healthcare facilities. A narrative report was prepared and used alongside the quantitative findings to inform the ToC workshops and identify areas requiring further exploration in the qualitative study.

### ToC workshops

A more detailed account of the process is provided in the protocol [28]. Three ToC workshops were conducted with mental health stakeholders responsible for policy-making/ coordinating mental health services health administrators/and coordinators) and the implementers (healthcare providers, heads of facilities, and mental health experts). The participants (Table 3) were selected purposively based on their mental health service provision roles. The study team appreciated and tried to engage MHS service users and their relatives; however, the MHS user organization still needed to be established in the district**.** The first ToC workshop was conducted with mental health specialists and researchers, where the provisional ToC for integrated care for people with AUD was mapped. The provisional TOC described the desirable goals of integrated AUD, mapped possible pathways to achieve these goals, and identified necessary pre-conditions based on the synthesized evidence. Then two ToC meetings were carried out with mental health stakeholders at the national, regional, and district levels (including mental health coordinators at the national, regional, and district levels, representatives from the district and region health management teams, and general health workers)**.** These two ToC meetings led to further refinement and development of the ToC map to integrate care for people with AUD.

**Table 3:** ToC workshop participants

Stakeholders group	Female	Male	Total
Mental health expert	5	4	9
Healthcare providers and heads of facility	12	6	18
Health administrators/coordinators	5	7	12

The ToC meetings were conducted from October 2020 to May 2021 and facilitated by D.M. The discussions were conducted in Swahili and English, audio recorded, and the study coordinator took notes. The discussions took around 2.5 to 3 h.

The notes and audio recordings were reviewed and combined to develop and refine the draft ToC map.

### Qualitative study

The draft ToC map of an adapted model for integrating intervention for AUD in PHC was presented to participants. We used semi-structured interviews and focus group discussions (FGDs) with mental health stakeholders to assess the anticipated feasibility, acceptability, potential facilitators and barriers, and implementation strategies of the adapted integrated care model. The participants were selected purposively based on their mental health service provision roles. Involves representatives from the community, facility, and organization. Due to their role and position, the heads of facilities and the district, regional, and national mental health coordinators participated in both ToC workshops and qualitative (Table 4).

**Table 4:** Qualitative study participants

	Participant	Individual interview (n) = number of participants	FGD (N) = number of participants
Community	Community leader	2	
	Religious representative	2	
	Women’s group leader	1	
Facility	Health care providers		20
	Head in charge		6
Organizational	District	3	
	Region	2	
	National	1	

*FGD* Focus Group Discussion

A total of 11 interviews and three FGDs were conducted. All interviews and FGDs were conducted in Swahili and audio-recorded. The interviews lasted 60 to 90 min, whereas the FGD lasted 2 to 2 0.5 h. The interviews and FGDs were transcribed verbatim and then translated into English. The translated transcripts were imported into Nvivo-12 for data analysis. Thematic analysis was used.

### Triangulation of the data

Finally, the data from the three methods were triangulated to develop the adapted model for integrating interventions for AUD in PHC.

### Ethical approval

The study was reviewed and approved by the institutional review boards (IRB) of Addis Ababa University College of Health Science in Ethiopia (protocol number: 023/19/psyc) and the Muhimbili University of Health and Allied Sciences in Tanzania (Ref.No.DA.282/298/01.C/). All methods were carried out in accordance with relevant guidelines and regulations. Informed consent was obtained from all participants in the different sub-studies.

## Results

A situational appraisal revealed resources and system functioning. The Theory of Change proposed an intervention package to support integrating mental health services, including AUD, at the community, facility, and organization. We used the WHO health system building blocks framework to summarize the results [34] (i) service delivery, (ii) health workforce, (iii) health information systems, (iv) access to essential medicines, (v) financing, and (vi) leadership/governance.

i) Service delivery

Situational appraisal revealed that the community infrastructure, such as community-based rehabilitation, self-help groups, and non-government organizations to support mental health care in the community, was unavailable. Mental health care, including AUD services, was delivered at the facility through general outpatient clinics, and inpatient care was unavailable. In the ToC, the proposed community interventions were (i)community awareness-raising (for example, providing health education and use leaflets), (ii) identification of people with problematic alcohol use and supporting a continuum of care, (i) Community awareness-raising aimed to (a) increase knowledge about the presenting features and associated consequences of problematic alcohol use and AUD and (b) increase health-seeking behavior and available treatment. The interventions will be conducted by distributing information leaflets and posters, providing health education in social and economic community gatherings, and other community health-related awareness-raising campaigns. In the meantime, it was recommended to integrate AUD services within the existing home-based HIV service. At the facility, they were (i) Training for HCPs; the package proposes to conduct training for at least seven days, combining theoretical and practical sessions to help HCPs with basic theoretical knowledge about problematic alcohol use, AUD, and associated physical and mental health conditions and to gain skills for AUD management. The WHO Mental Health Gap Action Programme (mhGAP) training manual [35] will be adapted such that Tanzanian case scenarios and examples will be used for practical and discussion sessions. The WHO manuals for AUD Identification Test (AUDIT) [36] and brief intervention packages for hazardous and harmful drinking [37] will also be used during training. Training will help HCPs improve knowledge and skills to; (a) detect people with AUD using user-friendly AUD screening tools, (b) prescribe and monitor medications for AUD such as acamprosate, disulfiram, and other psychotropic medication, (c) offer basic psychological interventions, for example, motivational intervention, (d) provide basic psychosocial support (addressing stressors and increasing social support), and (e) provide referral should there be an indication and making a follow-up care plan. (ii) Supportive supervision: the aim is to improve the capacity of the HCPs to provide care for people with AUD and address factors that support and affect their provision of services. A supportive supervision plan for mental health, including AUD, will be integrated into the existing facility’s supervision plan.

ii) Health workforce

A situational appraisal found that the district does not have community health workers (CHWs) to provide health services in the community, even though they are in the Ministry of Health’s human resource structure and plans. The number of HCPs in healthcare centers ranged from 13 to 22, most of whom were nurses. Approximately one HCP served 570 to 2250 population. Therefore, mental health care, including AUD, was delivered by non-specialized practitioners. In ToC workshops, the intervention packages propose that CHWs implement the interventions as per the Ministry of Health services organization plan. It was proposed that lay HCPs currently carrying out home-based care for HIV services will receive training and be incentivized to do these interventions. Moreover, the organization to improve recruitment for HCPs and build their capacity to detect and manage AUD.

iii) Health information systems

A situational appraisal revealed that health management information systems (HMIS) do not have sufficient information to capture mental health conditions, including AUD. The intervention packages ask the management team at the district, regional, and national to improve the HMIS to capture sufficient information for integrating mental health conditions, including AUD.

iv) Access to essential medicine

Compared to the need, an appraisal revealed insufficient medications for treating common mental disorders, including AUD. The intervention packages ask the district, region, and national management team to improve the supply and management of essential medication and medical equipment for delivering health services, including AUD.

xxii) Financing

It was observed that financing and resources for mental health services, including AUD, were insufficient to meet needs. Therefore, the intervention package asks the organization to improve the funding of mental health services, including AUD. The proposed strategies include advocating and sensitizing stakeholders to support the management of interventions for the integrated model for AUD.

vi) Leadership/governance

**Fig. 2:**
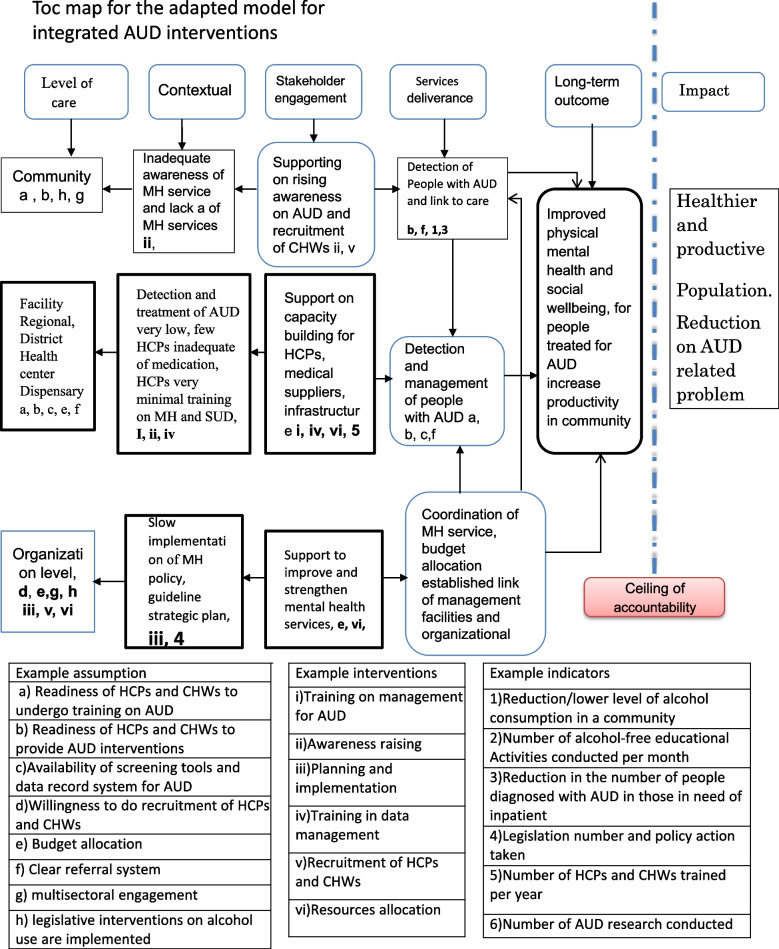
TOC map for the adapted model for integrated AUD interventions

The situational appraisal revealed that the council health management (CHMT) organizes mental health services in the district. The coordination and implementation of mental health services, including AUD services, are guided by the national health policy [38], national health sector strategic plan V [26], the mental health act (2008) [39], mental health regulations (2012), and the national strategic plan for non-communicable diseases [27]. The district mental health coordinator is one of the members of the CHMT and coordinates the implementation of mental health services in the district. The ToC intervention asks the district CHMT to support training for CHWs. Through training, CHWs will learn skills to identify people with problematic alcohol use, link them to the facility, and support a continuum of care. The package asks the district organization to recruit CHWs and train them to identify people with mental health conditions, including AUD. The district mental health coordinator should engage the NGOs supporting home-based HIV services in the community to support AUD services. The ToC intervention packages at the organization level include (i) strengthening infrastructure for the mental health care delivery system, (ii) ensuring the availability of human resources, and (iii) improving the HMIS, medical supplies, supportive supervision, and financing of mental health care. The proposed strategies to achieve these include (i) improving recruitment for HCPs and building their capacity to detect and manage AUD and (ii) Engaging with stakeholders to support the implementation of the AUD intervention packages (for example, for awareness-raising, development, and implementation of the policy guidelines, legislative, and strategic plans), and (iii) advocating and sensitizing stakeholders to support the management of interventions for the integrated model for AUD (Table 5) and Fig. 2.

**Table 5:** Summary of intervention and expected short-term outcomes of adapted model for integration of intervention for AUD

Level of HCS	Main objective	Intervention packages	Expected short-term outcomes
Community	Detection of people with AUD and link to care	Awareness-raising, improving access to care, engaging d community Training for CHWs	Improve need, access, and engagement to AUD services
Facility	Identify and manage people with AUD	Training HCPs, supportive supervision, and improve infrastructures for the provision of mental health services	Competent AUD services Increase detection and management of people with AUD Sustainable AUD service delivery
Organizational (district, region, and national)	improve and strengthen mental health services	supporting infrastructure for service delivery, ensuring the availability of staff, improving medical supplies and management financing mental health services	Sustainability of integrated AUD services Improve accessibility and coverage for AUD services Improve ownership and engagement in managing AUD services

### Feasibility and acceptability of the adapted model

We conducted 11 individual interviews and three FGDs with 26 participants (Table 3). Generally, the qualitative study findings indicated that the adapted model for the integration of AUD was perceived to be feasible and acceptable. However, participants reported some barriers and facilitators that must be overcome. Below is a description of the themes and participants’ quotes for the feasibility, acceptability, barriers, facilitators, and strategies to overcome them.

#### Adapted model as a structural tool to the existing provision of the MHS

Participants considered that the adapted model for integrating interventions for AUD would be possible to implement. Facilitators included the recognition of the relevance of mental health within PHC, the involvement of stakeholders in the adaptation process, the use of existing health system resources, and the existence of structured plans to deliver intervention packages.

*I think the adapted model will be possible. We have been engaging in the process. The model provides a formal way of how services can be provided. Mental health is a primary health component, as has been provided with other health services in PHC. The adapted model will help us to be more organized (Participant 2, interview, representative from the organizational level)*

#### Shortage of providers

An inadequate number of providers in health care facilities and communities was reported to be among the barriers to implementing the community and facility intervention packages for the adapted model for integration of AUD in PHC.

*We are pushing to work long hours, attending to many patients. Sometimes you find the provider is providing more than one service simultaneously. If the current number of providers continues, implementing this plan will not be possible. (Participant 5, Head-in-charge FGD)*

#### Building the capacity of providers to manage people with AUD

Training HCPs, CHWs, and other lay-health care providers to have knowledge and skills to identify and manage people with problematic alcohol use was essential to providing competent care to people with problematic alcohol use. HCPs reported that this would motivate them and build their confidence to deliver care for people with AUD.

*If we have the knowledge and skills, we will confidently ask people about their alcohol use and other related problems. Most of us do not have that knowledge. At least we can manage those with psychosis. As they have a noticeable change in behavior (participant 7, HCPs’ FGD 1)*

#### Financing and prioritization of integrated mental health care

Participants reported that if the budget for overall mental health services, including AUD, was improved and if mental health was given priority, these would be essential facilitators of implementation. The current underfunding of mental health services was incompatible with delivering care.

*Most of the time, medications are not available in our facilities. The budget is a constraint. Most of the time, we can’t procure medicine at health facilities. But, the policy asks to provide mental health services for free. We end up giving patients referrals to the regional hospital; most of them can’t due to transport and other costs. (participant 4 HCPs’ FGD2)*

T*he government has put effort into other health issues; the same action should be set for mental health issues. As we speak today, there is little initiative for mental health. Therefore, more effort is needed (participant 3, HCPs’ FGD 1).*

## Discussion

This study aimed to adapt a model for integrating AUD interventions into the Tanzania PHC system. A study has revealed the resources, system functioning, and proposed intervention package to support integrating mental health services, including AUD, at the community, facility, and organization. Moreover, it’s the feasibility and acceptability of integrating these interventions into PHC.

The proposed interventions, community awareness-raising, identification of people with AUD, and training of community and health care providers to facilitate the delivery of services for the integration of the AUD interventions in the community and facility aligned with a previous review of strategies to facilitate integrated care for people with alcohol problems [40]. Also, the study revealed limited resources and system functioning for delivering services for integrating AUD interventions in the community, similar to another study in Eastern Africa [20].

The appraisal revealed that compared to the need, the district has a minimal number of the health workforce to support integrating AUD interventions in PHC in the community. This is similar to findings from other studies in SSA [20, 23, 24]. Notably, the intervention packages propose that the organization improve recruitment and consider the task-shifting model. These align with the recommendation and strategies from the Grand Challenges: Integrating Mental Health Care into the Non-Communicable Disease (NCD) Agenda recommendation [41], and review of strategies to facilitate integrated care for people with an alcohol problem [40]

Similar to another study in Eastern Africa [20], the study found that the health management information systems (HMIS)do not capture sufficient information about integrating mental health conditions, including AUD. Of note is that the proposed intervention asks the management to improve the HMIS to facilitate the integration of the AUD intervention, similar to a systematic review of global evidence for strategies to facilitate integrated care for people with alcohol problems [40].

In keeping with previous studies in SSA [20, 23, 24], the findings revealed insufficient medication for treating common mental disorders, including AUD. The results reflect the recommended strategy accorded to expanding the availability of medical supply by the WHO Mh-gap program [19] to facilitate the integration of care for the priority mental and neurological conditions, including AUD, to reduce the treatment gap.

Financing and resource allocation for mental health, including AUD, do not meet the need of other SSA studies [20]. The intervention package asks the management to improve the budget and resources for supporting the integration of the AUD interventions, aligning with a previous review on strategies to facilitate integrated care for people with alcohol problems [40] and the Grand Challenges review recommendation on integrating MHC into the NCD Agenda [41].

In keeping with the WHO building blocks of health [34] that emphasize effective governance and leadership should ensure the existence of the strategic plan, policy, and frameworks, the study revealed that strategic plans and guidelines guide the organization and implementation of mental health services, including AUD, in the study area. Also, the intervention package asks the organization's management to continue coordinating the implementation of the integrated AUD intervention packages, likewise another study in Eastern Africa [20].

In a qualitative study, generally, participants considered that the AUD intervention packages could be integrated into PHC, similar to other studies in SSA [20, 23, 24]. However, participants reported the following factors facilitating the integration of the AUD intervention in PHC, use of the existing health system resources, and the existence of structured plans to deliver the intervention, similar to another study in Eastern Africa [20] and building the capacity of providers to manage people with AUD likewise other studies in SSA [20, 23, 24], financing, and prioritization of the MHS, similar to another study in Eastern Africa [20] and systematic reviews on barriers and facilitators for integrating mental health in PHC [42]. These factors are in keeping with a review of the provision of mental health care to cancer patients [43]. On the other hand, a shortage of providers was reported to hinder the integration of AUD intervention, similar to other studies that reported integrating mental health in non-communicable diseases [44].

### Implications

Adapting a model for integrating interventions for AUD in Tanzanian PHC responded to the WHO call to expand access to mental health care and reduce the treatment gap (mhGAP) [19]). The WHO mhGAP guideline emphasizes the integration of evidence-based interventions for MNS, including AUD, in the general health care services to address the AUD treatment gap. However, the guideline has recommended that the health system’s contextual social-cultural factors be considered to enhance the effective, sustainable, acceptable, and feasible adapted integrated AUD interventions in different socio-cultural and health system contexts. Therefore, our study team adapted a model for integrating AUD interventions in PHC in Tanzania, expecting to enhance the chances of scale-up and sustainability.

### Limitations

This study has limitations; we could not appraise some information on resources and system functioning with the potential to integrate interventions for AUD in PHC in the Moshi district council, such as Non-governmental and community-based organizations, traditional healers, and alternative healers. The reason is that a situational appraisal tool relies on data available in the public domain. We could not get opinions from the district's mental health service user organizations. Despite efforts to include them, they still need to be in the study area. The generalizability of the results could be limited because the study was conducted in the Moshi district council and used qualitative methods.

## Conclusion

The adapted model for the integrated AUD intervention in Tanzanian PHC revealed limited resources and system functioning for facilitating integrated AUD services. Nevertheless, it proposes the needed integrated AUD interventions as well as its barriers, facilitators, and strategies for overcoming them. There is a need to pilot the adapted model to inform plans for more comprehensive implementation or scaling up.

## Data Availability

All data used to write this paper is summarized in tables, figures, or within text in the article. Please get in touch with the corresponding authors should there be a need for this study.
